# Redox‐Active NiO_x_‐Catalyzed Li^+^ Capture‐Extraction Strategy for *t*BP‐Free Spiro‐OMeTAD Enables Exceptional Damp‐Heat Stability in Perovskite Solar Cells

**DOI:** 10.1002/advs.202521825

**Published:** 2026-01-26

**Authors:** Yun Seop Shin, Minjin Kim, Jaehwi Lee, Chang Hyeon Yoon, Jongdeuk Seo, Gyeong‐Cheon Choi, Sujung Park, Min Jung Sung, Kyungnan Son, Sungjun Hong, Inyoung Jeong, Junseop Byeon, Yimhyun Jo, Dongmin Lee, Minseong Kim, Shinuk Cho, Ji‐Youn Seo, Jin Young Kim, Dong Suk Kim, SeJin Ahn

**Affiliations:** ^1^ Graduate School of Carbon Neutrality Ulsan National Institute of Science and Technology (UNIST) Ulsan Republic of Korea; ^2^ School of Energy and Chemical Engineering Ulsan National Institute of Science and Technology (UNIST) Ulsan Republic of Korea; ^3^ Photoenergy Research Center Korea Research Institute of Chemical Technology (KRICT) Daejeon Republic of Korea; ^4^ Ulsan Advanced Energy Technology R&D Center Korea Institute of Energy Research (KIER) Ulsan Republic of Korea; ^5^ Department of Nano Fusion Technology Pusan National University Busan Republic of Korea; ^6^ Department of Physics and Energy Harvest Storage Research Center University of Ulsan Ulsan Republic of Korea; ^7^ Photovoltaics Research Department Korea Institute of Energy Research (KIER) Daejeon Republic of Korea

**Keywords:** dopant engineering, hole‐transporting layer, nickel oxide, perovskite solar cells, spiro‐OMeTAD

## Abstract

For the spiro‐OMeTAD‐based hole‐transporting layer (HTL), despite its widespread implementation, the pragmatic deployment of perovskite solar cells remains profoundly constrained by multifaceted intrinsic challenges—most notably the inclusion of *t*BP and the deleterious migration behaviors of Li^+^ ions—which collectively undermine long‐term thermal stability, as even advanced encapsulation schemes fail to arrest irreversible performance degradation. Here, we introduce a redox‐catalytic strategy that enables simultaneous Li^+^ capture and extraction from the spiro‐OMeTAD solution, achieving a *t*BP‐free, Li^+^‐free dopant system. Micro‐sized nickel oxide (NiO_x_) powder act as a redox‐active catalyst, inducing ultrafast oxidation of spiro‐OMeTAD through a redox cascade mechanism while promoting LiTFSI solvation and Li^+^ sequestration. The reacted NiO_x_ particulates, along with sequestered Li^+^ ions and Li‐related byproducts, are completely removed during filtration, yielding a purified dopant formulation devoid of instability‐inducing residues. As a direct outcome, the NiO_x_‐catalyzed HTL furnishes an outstanding powder conversion efficiency of 25.24%, commensurate with that of devices employing conventionally doped HTLs containing *t*BP and LiTFSI. More importantly, the concomitant removal of pernicious constituents imparts exceptional operational resilience, with the device retaining over 95% of its initial efficiency after 1,000 h under stringent damp‐heat stress.

## Graphical abstract

The redox‐active nature of NiO_x_ enables ultrafast oxidative doping of spiro‐OMeTAD within seconds. Concurrently, NiO_x_‐Li^+^ coordination complexes formed during this redox process are selectively removed by filtration, effectively extracting residual Li^+^ ions from the film. The absence of *t*BP and Li^+^ ions underpins exceptional damp‐heat stability of the resulting devices.

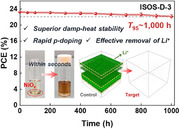

## 1 Introduction

The strategic integration of the *π*‐conjugated small molecule spiro‐OMeTAD, in conjunction with 4‐*tert*‐butylpyridine (*t*BP) and p‐dopants such as lithium bis(trifluoromethanesulfonyl)imide (LiTFSI), has emerged as a cornerstone in the rational design and optimal operation of hole‐transporting layer (HTL) systems [1, 2, 3, 4]. This cutting‐edge configuration underpins state‐of‐the‐art integrated platforms that consistently deliver unprecedented power conversion efficiency (PCE) in perovskite solar cells (PSCs) [5, 6, 7, 8], thereby representing an indispensable component of conventional PSC designs.

Despite its pervasive adoption, the practical deployment and eventual commercialization of PSCs remain encumbered by a constellation of formidable obstacles intrinsic to this system [9]. Among these, thermal instability issue has continued to be a subject of extensive discussion, as both the perovskite absorber and spiro‐OMeTAD HTL are vulnerable to thermal degradation. The perovskite layer decomposes thermally, releasing volatile products undergo that outgas and participate in irreversible consumption reactions. Recent studies, however, have demonstrated that, within tightly sealed, multiple‐barrier encapsulation architectures [10, 11, 12], such degradation can proceed in a quasi‐reversible manner by suppressing volatile outgassing and confining reaction equilibria within a closed system. Nevertheless, even under these stringent encapsulation conditions, once the constituent layers undergo thermally induced chemical degradation or a decline in their functional properties, the prevention of efficiency losses becomes practically unattainable.

In spiro‐OMeTAD‐based HTL systems, the pervasively employed *t*BP intrinsically compromises the structural and electronic integrity of the hole conductors through irreversible degradation pathways [13, 14, 15, 16, 17]. Consequently, under elevated thermal stress, even tightly encapsulated architectures become ineffectual in preserving operational stability, rendering the protective function of such encapsulation schemes fundamentally limited. While the thermal instability of spiro‐OMeTAD‐based HTL systems has been predominantly ascribed to the incorporation of *t*BP, thereby driving intensive efforts toward the development of *t*BP‐free and/or *t*BP‐substituted additive schemes, an equally pivotal yet frequently overlooked factor stems from the intricate Li^+^ ion migration phenomena within the device [18]. Migrated Li^+^ ions perturb both the electronic structure and intrinsic charge distribution of the perovskite [19], while their lattice incorporation engenders micro‐strain [20], compromising the structural integrity of the perovskite phase and facilitating phase transition [21]. Such migration of Li^+^ ion exerts profound deleterious effects on the subjacent perovskite layer, a process that is further potentiated under elevated thermal stress.

Accordingly, the most viable strategy for fortifying both the structural and functional resilience of HTL under elevated thermal stress entails the deployment of a *t*BP‐free dopant framework, concomitantly complemented by strategies to sequester Li^+^ ions following the oxidation of spiro‐OMeTAD and thereby mitigate their migratory behavior. This strategy inherently engenders a solubility constraint of LiTFSI in the absence of *t*BP, thereby necessitating the introduction of auxiliary additives that can concurrently facilitate solvation enhancement and Li^+^ sequestration through specific coordination interactions with Li^+^ ions. In this context, 12‐crown‐4 [22], olelyamine‐functionalized nickel oxide (NiO_x_) nanoparticle [23], and ethylene carbonate [24] have been reported as representative additives capable of fulfilling these dual functionalities with pronounced efficacy. However, the sequestered Li^+^ ions are prone to persist within the solution or film in conjunction with the coordinating additives, and the consequent implications of these residual Li^+^‐additive complexes have not yet been comprehensively delineated. Such remnant ionic species may still act as latent degradation precursors, thereby underscoring the imperative for devising extraction strategies to effectively eliminate them from the HTL matrix.

Herein, we presented a novel approach that achieves both Li^+^ capture and subsequent extraction from the *t*BP‐free spiro‐OMeTAD solution through the strategic integration of micro‐sized NiO_x_ powder as a redox‐active catalyst. Our approach leverages the inherent redox activity of NiO_x_ to enable ultrafast hole injection via a redox cascade mechanism, achieving complete oxidation in a sub‐5‐second time frame while concurrently enhancing electrical conductivity. Following the ultrafast hole injection kinetics, the negatively charged NiO_x_ enhances LiTFSI solvation efficacy, enabling its dissolution in *t*BP‐free formulations. By intentionally employing micro‐sized NiO_x_ powder that can be readily intercepted during filtration, the reacted NiO_x_ particulates and sequestered Li^+^ ions are simultaneously eliminated from the solution. This approach yields a benign spiro‐OMeTAD formulation devoid of *t*BP, Li^+^ ions, and remnant NiO_x_, all of which are detrimental to long‐term stability. As a result, PSCs incorporating this multifunctional dopant system achieved an impressive PCE of 25.24%. Furthermore, the concomitant removal of deleterious constituents, in concert with the elevated *T*_g_ of the doped spiro‐OMeTAD, collectively conferred endurance exceeding 1,000 h under prolonged damp‐heat stress.

## 2 Results and Discussion

**FIGURE 1:**
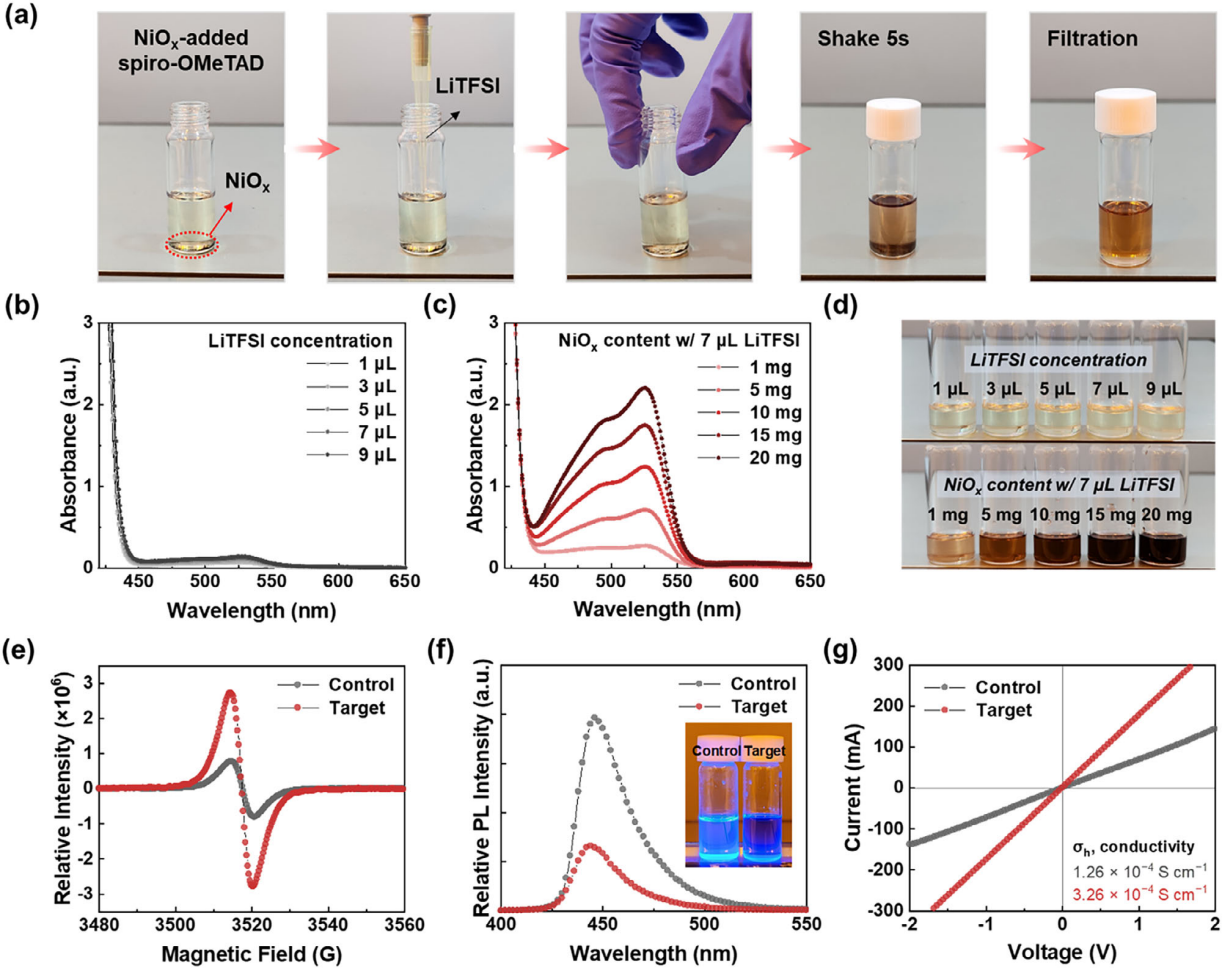
Optical and electrical characteristics of NiO_x_‐mediated p‐doped spiro‐OMeTAD (a) Schematic illustration of the NiO_x_‐mediated p‐type doping process in the spiro‐OMeTAD solution. Absorption spectra of spiro‐OMeTAD solutions (b) with varying concentrations of LiTFSI in the absence of NiO_x_ and (c) with different amounts of NiO_x_ powder in the presence of 7 µL of LiTFSI. (d) Photographic images of the corresponding spiro‐OMeTAD solutions under each condition. (e) ESR and (f) PL spectra of spiro‐OMeTAD solutions under control and target conditions. (g) *I*‐*V* characteristics of hole‐only devices (FTO/spiro‐OMeTAD/Au) used to evaluate the conductivity of the spiro‐OMeTAD films.

Figure 1a depicts the schematic illustration of the doping process of NiO_x_‐integrated spiro‐OMeTAD in the *t*BP‐free formulation. No discernible color change was observed between the spiro‐OMeTAD solution and the micro‐sized NiO_x_ powder; however, upon the addition of the LiTFSI dopant, a distinct color transition to a reddish hue was evident within 5 s upon simple mixing, indicative of spontaneous oxidation. This oxidation phenomenon in the spiro‐OMeTAD solution was corroborated by the appearance of an absorption peak at 520 nm. In the absence of NiO_x_ powder, however, the absorption intensity at 520 nm remained unchanged, even upon increasing the quantity of the LiTFSI dopant (Figure 1b). Meanwhile, increasing the quantity of NiO_x_ powder markedly facilitated the spontaneous oxidation of spiro‐OMeTAD under conditions involving a quantity of LiTFSI (7 µL), achieving complete oxidation within 5 s and even in the absence of light illumination, as substantiated by the pronounced enhancement in the absorption intensity at 520 nm [25, 26], characteristic of spiro‐OMeTAD^•+^TFSI^−^ (Figure 1c). Alongside the variation in absorption intensity, a distinct color change in evidently depicted in Figure 1d. In the *t*BP‐free formulation, the limited solubility of the LiTFSI dopant impeded the spontaneous p‐doping process, even under exposure to light, as illustrated in Figure S1. In this study, the spiro‐OMeTAD solution containing 7 µL of LiTFSI was designated as the control‐HTL, whereas the formulation comprising 7 µL of LiTFSI and 3 mg of NiO_x_ powder was referred to the target‐HTL, both of which were prepared without *t*BP. These doping conditions were meticulously optimized, considering that excessive doping levels can inversely perturb the intrinsic electrical conductivity of spiro‐OMeTAD [27, 28] and thereby compromise the ensuring device efficiency (Figure S2 and Table S1).

The absorption spectra provided conclusive evidence for the stabilization of spiro‐OMeTAD^•+^TFSI^−^ radical species, which was further substantiated by electron spin resonance (ESR) measurements. Under the target‐HTL condition, a higher concentration of positive radicals manifested as an intensified ESR signal (Figure 1e). Additionally, their presence also induced pronounced exciton quenching by polaron‐mediated charge transfer, thereby culminating in a markedly reduced photoluminescence (PL) intensity (Figure 1f). Ultimately, as shown in Figure 1g, the increased dopant‐mediated hole carriers led to a 2.5‐fold enhancement in the electrical conductivity of spiro‐OMeTAD, augmenting from 1.26 × 10^−4^ to 3.26 × 10^−4^ S cm^−1^. It is evident that the *t*BP‐devoid control‐HTL system exhibits intrinsically constrained p‐doping efficacy, stemming from the attenuated electrical conductivity induced by the poor solubility of LiTFSI. Collectively, these findings validate the NiO_x_‐integrated doping paradigm as a transformative approach that transcends the inherent doping limitations of *t*BP‐free control formulations.

**FIGURE 2:**
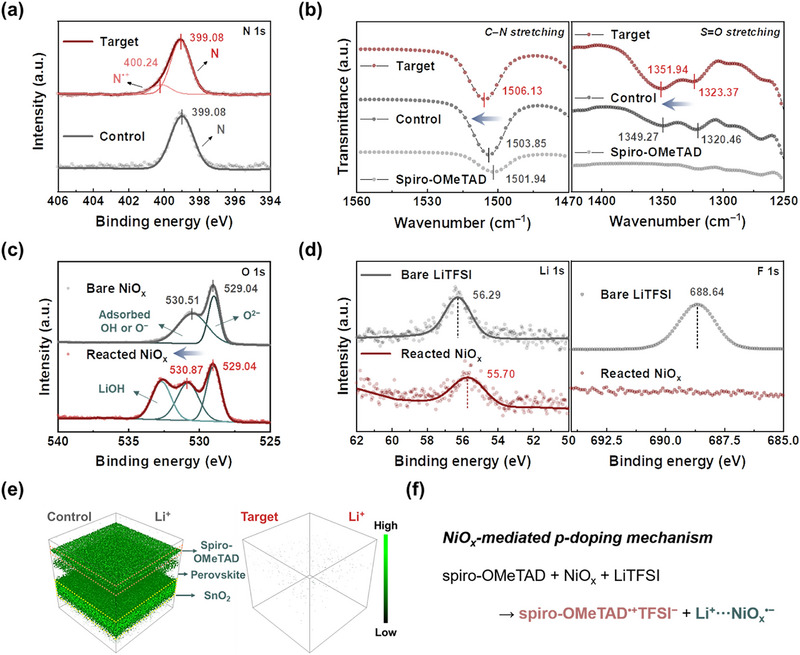
Proposed mechanism of NiO_x_‐mediated p‐doping in spiro‐OMeTAD (a) XPS spectra corresponding to N 1s for control‐ and target‐HTLs. (b) FTIR spectra of pristine spiro‐OMeTAD, control‐HTL, and target‐HTL. (c) XPS spectra corresponding to O 1s for pristine and LiTFSI‐reacted NiO_x_ powders. (d) XPS spectra corresponding to Li 1s (left) and F 1s (right) for pristine LiTFSI and reacted NiO_x_ powders. (e) 3D ToF‐SIMS images showing the distribution of Li^+^ ions in FTO/SnO_2_/perovskite/control‐ and target‐HTL device architectures. (f) Schematic illustration of the proposed p‐doping mechanism in NiO_x_‐mediated spiro‐OMeTAD HTLs.

The spontaneous pre‐oxidation of spiro‐OMeTAD serves as a critical prerequisite for the subsequent stabilization of spiro‐OMeTAD^•+^ radical cations [29, 30]. To validate the occurrence of this process in the presence of NiO_x_, X‐ray photoelectron spectroscopy (XPS) analyses were conducted on both the control‐ and target‐HTLs. As depicted in Figure 2a, the control‐HTL exhibited a binding energy peak at 399.08 eV, attributed to neutral nitrogen species, whereas the target‐HTL revealed an additional broadened peak at 400.24 eV, corresponding to the oxidized N^•+^ state. The observed peak broadening signifies a shortened transverse relaxation time, likely originating from rapid electron exchange between the formed spiro‐OMeTAD^•+^ species and their neutral counterparts, thus substantiating the efficient occurrence of the pre‐oxidation process [31]. Subsequent to the pre‐oxidation process, the stabilization of spiro‐OMeTAD^•+^ positive radicals is facilitated by Coulombic interactions with TFSI^–^ anions. To gain a comprehensive insight into the charge compensation kinetics between spiro‐OMeTAD^•+^ species and TFSI^–^ anions, Fourier‐transform infrared spectroscopy (FTIR) measurements were performed under both control‐ and target‐HTL conditions (Figure 2b). The C–N stretching vibration of the triphenylamine moiety in pristine (undoped) spiro‐OMeTAD appeared at 1501.94 cm^−1^. Upon doping, this vibrational mode exhibited a progressive shift toward a higher wavenumber to 1503.85 and 1506.13 cm^−1^ for the control‐ and target‐HTL conditions, respectively, highlighting the more efficient generation of radical species facilitated by the NiO_x_‐based doping strategy [32]. Concurrently, the enhanced electrostatic binding in the target‐HTL condition manifested as a discernible shift of the S = O sulfonamide stretching vibration in TFSI^–^ anions toward higher wavenumbers.

Within the framework of the conventional doping mechanism, it is imperative to delineate the mechanistic contribution of NiO_x_ powder, specifically, whether it functions analogously to molecular O_2_, *t*BP, or serves a dual role mimicking both. First, to rigorously eliminate the involvement of O_2_, the doping kinetics of the spiro‐OMeTAD solution were systematically investigated under both ambient conditions and an inert nitrogen‐filled glovebox environment (Figure S3a). Notably, comparable doping efficiencies of spiro‐OMeTAD were attained under both ambient and inert conditions with same quantities of LiTFSI and NiO_x_, suggesting that NiO_x_ powder actively participates in the pre‐doping process independent of molecular O_2_ involvement (Figure S3b,c). Second, the solvation capability of NiO_x_ powder toward the LiTFSI dopant in the spiro‐OMeTAD solution was investigated in the absence of the *t*BP additive (Figure S4). Unexpectedly, the incorporation of NiO_x_ powder facilitated the dissolution of the LiTFSI dopant, thereby triggering the spontaneous p‐doping process of spiro‐OMeTAD, as corroborated by the intensified ESR signal (Figure S5). Consequently, these findings indicate that NiO_x_ powder exhibit dual functionality, effectively substituting for both molecular O_2_ and the *t*BP additive within the conventional doping framework.

To gain a comprehensive understanding of the chemical interactions between NiO_x_ and LiTFSI, XPS analyses were meticulously conducted on both pristine NiO_x_ and NiO_x_ powder precipitated from the spiro‐OMeTAD solution. As depicted in Figure S6, quantitative analysis of the Ni^2+^/Ni^3+^ ratios revealed a pronounced increase from 0.694 for the pristine NiO_x_ powder to 0.976 for the precipitated NiO_x_ powder, providing clear evidence that Ni^3+^ species were partially reduced to Ni^2+^ during the oxidative doping of spiro‐OMeTAD. This redox process is accompanied by a modulation of the surface electronic structure of NiO_x_, leading to an increased Lewis basicity of the surface oxygen species. Given that the TFSI^–^ anion is a weakly coordinating counterion owing to its highly delocalized charge distribution, Li^+^ ions readily dissociate from LiTFSI and preferentially engage in coordination with these increasingly Lewis‐basic oxygen sites on the NiO_x_ surface. Such coordination interaction is substantiated by XPS analysis. Specifically, while the binding energy associated with lattice oxygen remained invariant at 529.04 eV, the feature corresponding to chemisorbed non‐lattice oxygen species exhibited a discernible shift from 530.51 to 530.87 eV, indicative of a modified chemical environment (Figure 2c). In parallel, the Li 1s XPS spectrum displayed a systematic shift toward lower binding energy compared to that of the pristine LiTFSI precursor (left in Figure 2d). Taken together, the correlated shifts in the non‐lattice oxygen and Li 1s features unambiguously demonstrate that non‐lattice oxygen species act as Lewis base sites that mediate the chemical coordination of Li^+^ ions by the NiO_x_ powder during the doping process. Additionally, LiOH‐related signals are also observed in the precipitated NiO_x_ powder, which can be attributed to Li^+^ hydration reactions [15, 33] (Figure 2c and Figure S7). This interpretation is further corroborated by the complete absence of an F 1s signal in the XPS spectrum of the precipitated NiO_x_ powder (right in Figure 2d), which provides compelling evidence for the complete dissociation in the presence of NiO_x_. Such robust coordination between surface oxygen moieties of NiO_x_ and Li^+^ ions effectively enhances the solvation capability toward the LiTFSI dopant, thereby mitigating dopant precipitation in the spiro‐OMeTAD solution and enabling stable dissolution even in the absence of *t*BP.

By virtue of the inherent powder size of NiO_x_, it is selectively removed during the filtration process; moreover, the subsequent coordination between NiO_x_ and Li^+^ ions facilitates the exclusion of deleterious Li^+^ species from incorporation within the spiro‐OMeTAD film. To ensure the effective removal of incorporated NiO_x_ powder and Li^+^ ions, we deliberately employed micro‐sized NiO_x_ powder, which was subsequently eliminated during the filtration step in device fabrication. Three‐dimensional (3D) time‐of‐flight secondary ion mass spectroscopy (ToF‐SIMS) imaging provided a direct visualization of the spatial distribution of Li^+^ ions within the devices (Figure 2e and Figure S8). In the control‐HTL film, Li^+^ ions exhibit a non‐uniform distribution and are chiefly localized at the Au/HTL/perovskite and perovskite/SnO_2_ interfacial regions (left in Figure 2e). In contrast, the coordination between NiO_x_ and Li^+^ ions effectively scavenged residual Li^+^ ions in the target‐HTL film (right in Figure 2e), and the resulting Li^+^‐containing NiO_x_ powder was subsequently removed during the filtration process. Quantitative evaluation based on the integrated Li^+^‐related ToF‐SIMS spectral intensity reveals that only approximately 2% of the initially introduced Li^+^ ions remain within the target‐HTL (Figure S9), unambiguously indicating that the vast majority of Li^+^ species are preferentially immobilized by NiO_x_ and subsequently excluded from the film during filtration. Collectively, the integration of the experimental findings in Figure S3, which confirm the role of NiO_x_ as a molecular‐O_2_‐independent electron‐accepting mediator, and the chemically elucidated interaction pathways described herein allows the redox mechanism to be systematically summarized in Figure 2f. The intrinsically redox‐active NiO_x_ promotes the oxidation of spiro‐OMeTAD through ultrafast hole injection, while its negatively charged surface selectively captures Li^+^ ions from the LiTFSI precursor, facilitating their dissolution even in the absence of *t*BP. The remaining TFSI^–^ anions then electrostatically stabilize the oxidized spiro‐OMeTAD^•+^ species, culminating in the formation of p‐doped spiro‐OMeTAD^•+^TFSI^–^ radicals. Subsequent solution filtration effectively removes all undesirable chemical species, including Li^+^ ions and NiO_x_, thereby enabling the deposition of a chemically innocuous spiro‐OMeTAD solution that contains only spiro‐OMeTAD^•+^TFSI^–^ radicals as the oxidation product.

**FIGURE 3:**
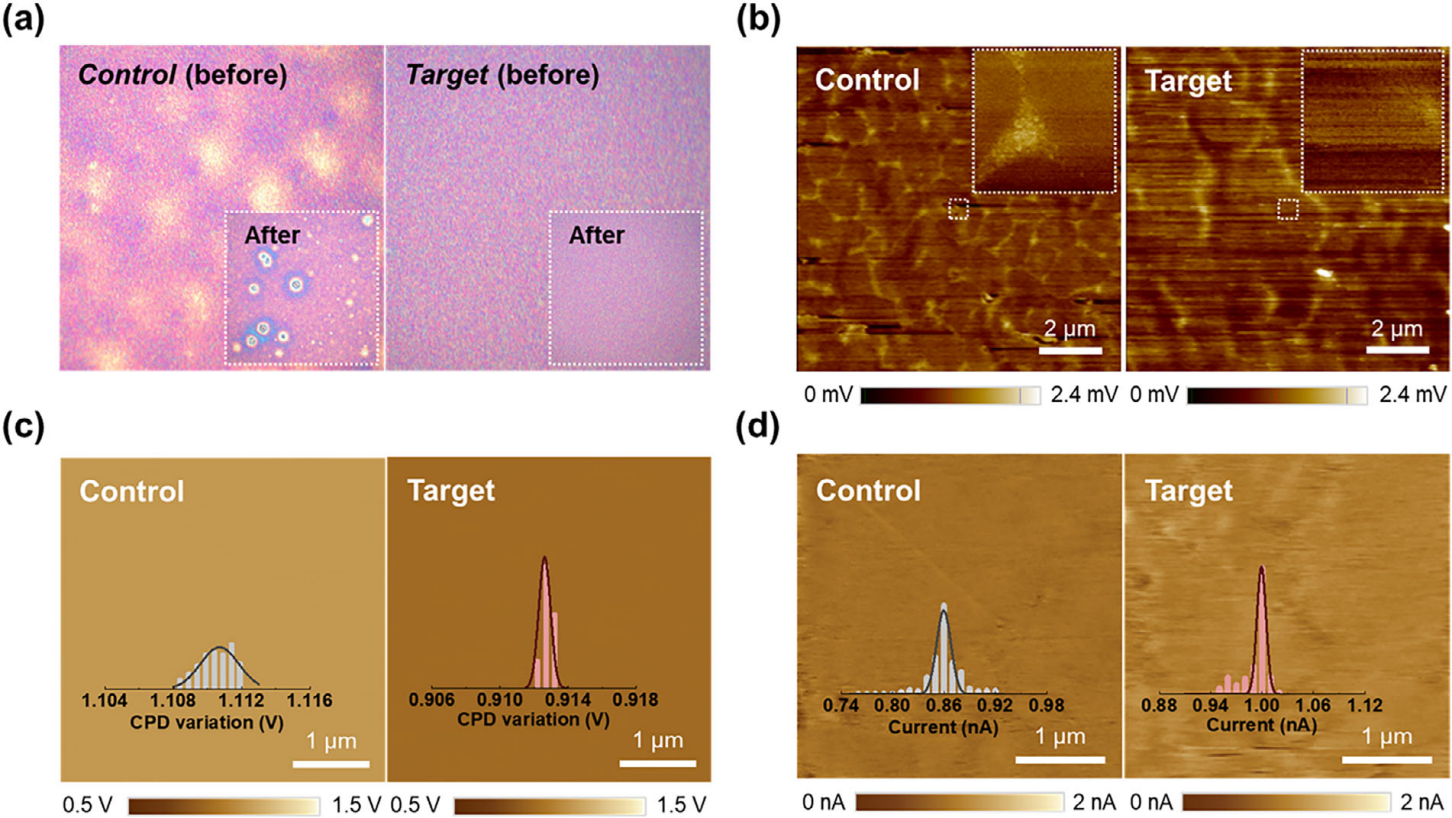
Morphological and interfacial characteristics of NiO_x_‐mediated p‐doped spiro‐OMeTAD films (a) OM images of control‐ and target‐HTLs before and after exposure to ambient conditions. (b) PiFM amplitude images of control‐ and target‐HTLs deposited on underlying perovskite films. PiFM amplitude imaging was conducted at 1508 cm^−1^, corresponding to the C–N stretching mode in the triphenylamine moiety, serving as a molecular fingerprint of spiro‐OMeTAD. (c) Surface potential images and corresponding distributions of the measured contact potential difference for the control‐ and target‐HTL films. (d) Conductive AFM images and corresponding current distributions of control‐ and target‐HTL films.

The inadequate solvation of the LiTFSI precursor within the spiro‐OMeTAD solution reportedly exerts a markedly deleterious effect on the morphological properties of the resulting film, particularly under *t*BP‐free doping conditions. The limited solubility of the dopant precursor renders it susceptible to precipitation during the spin‐coating process, leading to undesirable aggregation on the surface of the spiro‐OMeTAD film. The optical microscope (OM) images revealed pronounced precipitation of dopant phases in the control‐HTL film, in contrast, the target‐HTL film demonstrated a uniform and well‐defined morphology, free from undesirable dopant aggregation (Figure 3a). Upon storage under humid conditions, the control‐HTL film exhibited severe phase deformation accompanied by partially aggregated domains, likely stemming from the hygroscopic nature of LiTFSI [34] (inset in Figure 3a). In comparison, the target‐HTL film retained its superior morphological integrity under the same conditions, attributable to the enhanced solubility of the LiTFSI dopant and the selective sequestration of Li^+^ ions.

Furthermore, the absence of *t*BP in the formulation imposes constraints on the compositional homogeneity of the thin film [35], impeding the uniform spatial distribution of both dopant species and spiro‐OMeTAD molecules. To elucidate the potential impact of molecular inhomogeneity on charge transport from the perovskite layer, the spatial distribution of spiro‐OMeTAD molecules at the surface was further examined using photoinduced force microscopy (PiFM), a technique that enables localized infrared absorption mapping by detecting dipole–dipole interactions between the sample and an AFM tip. Specifically, PiFM amplitude images were collected at 1508 cm^−1^, corresponding to the C–N stretching vibration of the triphenylamine moiety, which serves as a molecular fingerprint of the spiro‐OMeTAD molecule (Figure 3b). Both control‐ and target‐HTL films were deposited atop the perovskite layer for comparative analysis. In the control‐HTL sample, the PiFM images revealed pronounced localized bright contrast, indicating spatial heterogeneity and possible aggregation of spiro‐OMeTAD molecules. Conversely, the target‐HTL sample exhibited a uniformly distributed amplitude signal with no discernible variation across the scanned area, suggesting a more homogeneous molecular distribution. Upon closer inspection, higher signal intensity was observed along the grain boundaries of the underlying perovskite layer in the control‐HTL, indicating preferential accumulation of spiro‐OMeTAD at these regions. Such non‐uniform molecular distribution at the interface is presumed to hinder hole extraction and facilitate non‐radiative recombination, ultimately leading to performance loss.

The intrinsic solubility constraints of the *t*BP‐free doping paradigm precipitate pronounced spatial inhomogeneity and concomitant anisotropic p‐type doping within the spiro‐OMeTAD film, potentially perturbing the uniformity of charge carrier transport and thereby compromising optimal device performance. To elucidate the impact of the NiO_x_‐mediated doping strategy on this issue, Kelvin probe force microscopy (KPFM) measurements were performed on both control‐ and target‐HTL films (Figure 3c). The target‐HTL film distinctly demonstrated a more spatially uniform surface potential profile and pronounced *p*‐type characteristics compared to the control‐HTL counterpart, underscoring the efficacious and homogeneous *p*‐type doping capability imparted by the NiO_x_ dopant across the entire spiro‐OMeTAD layer. Additionally, the modulation in surface potential imparted a distinctly enhanced p‐type character to the Fermi level (*E*_F_), shifting it from −5.02 to −5.21 eV, while concomitantly inducing a downward shift in the highest occupied molecular orbital (HOMO) energy level, thereby signifying an optimized hole transport energetics (Figure S10). The reduced energetic offset between the *E*_F_ and the HOMO energy levels, indicative of more effective p‐type doping, attenuates the interfacial barrier for hole extraction at the perovskite interface, thereby contributing to the mitigation of voltage losses. These favorable attributes were further substantiated by conductive atomic force microscopy (c‐AFM) analyses (Figure 3d), which revealed not only an overall enhancement in nanoscale conductivity but also a marked narrowing of the spatial distribution of current across the spiro‐OMeTAD layer. This signifies that the NiO_x_‐mediated doping strategy yields a more homogeneous network of conductive pathways, effectively suppressing localized insulating regions typically induced by incomplete or anisotropic doping.

**FIGURE 4:**
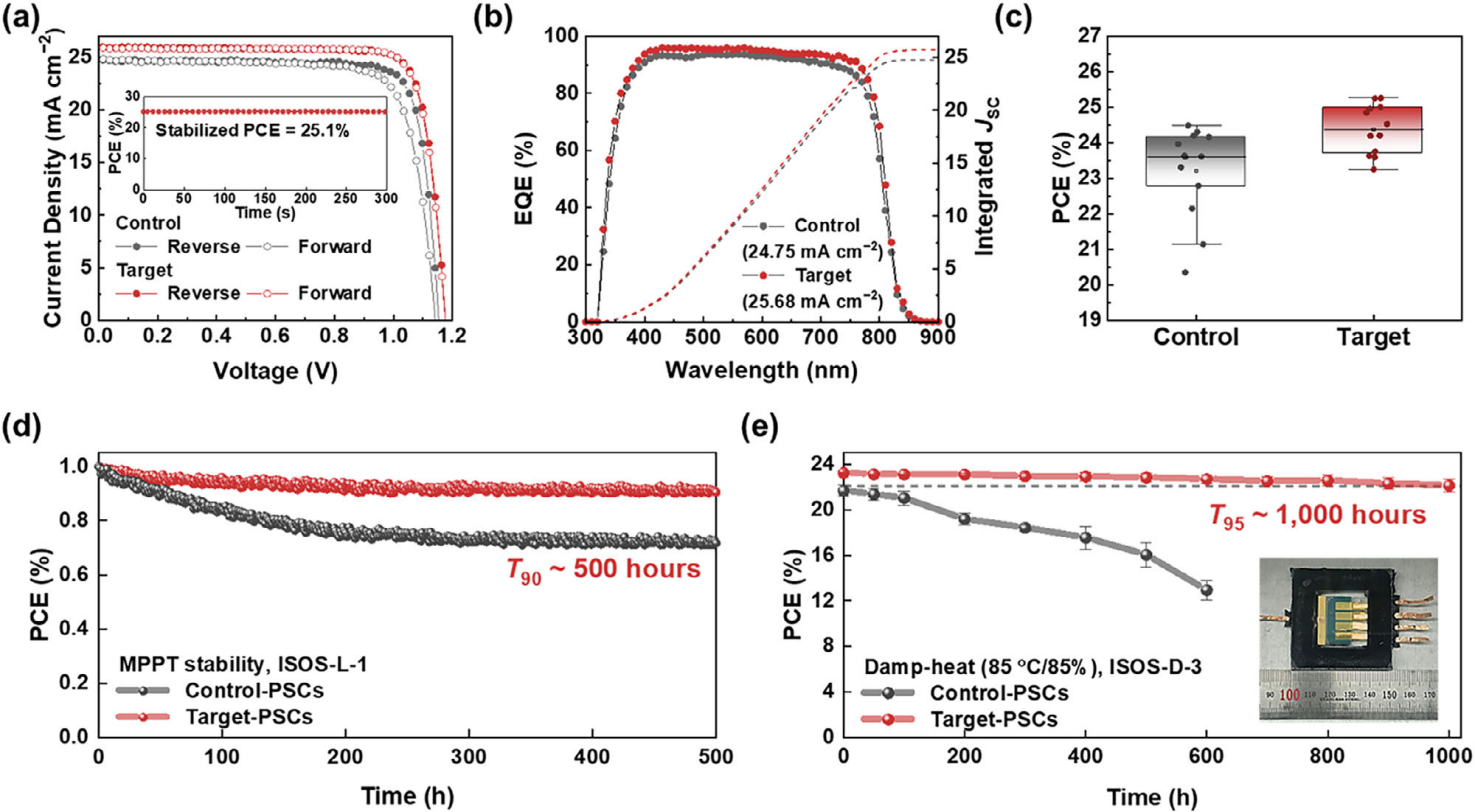
Photovoltaic performance of PSCs incorporating NiO_x_‐mediated p‐doped spiro‐OMeTAD HTLs (a) *J*‐*V* characteristics of control‐ and target‐PSCs measured under both reverse and forward scanning directions. Inset shows the SPO of the target‐PSCs. (b) EQE spectra and corresponding integrated *J*_SC_ for the control‐ and target‐PSCs. (c) Statistical distribution of PCE for the control‐ and target‐PSCs. (d) MPPT of encapsulated PSCs under continuous 1‐sun illumination. (e) Damp‐heat (85°C/85% RH) stability of the encapsulated PSCs.

Leveraging the aforementioned advantages of NiO_x_‐mediated doping strategy, we systematically evaluated the photovoltaic performance of devices constructed with the architecture: fluorine‐doped tin oxide (FTO)/tin oxide (SnO_2_)/formamidinium lead iodide (FAPbI_3_)‐based perovskite/spiro‐OMeTAD/Au. Under the target‐HTL conditions, the optimized PSCs achieved a maximum PCE of 25.24%, characterized by a short‐circuit current density (*J*_SC_) of 25.83 mA cm^−2^, an open‐circuit voltage (*V*_OC_) of 1.175 V, and a fill‐factor (FF) of 83.21%, surpassing the 23.60% attained with the control‐HTL conditions (Figure 4a and Table S2). A stabilized power output (SPO) of 25.1% was also recorded for the target‐devices under 1‐sun simulated illumination (inset in Figure 4a). Additionally, the integrated *J*_SC_ of 25.68 and 24.75 mA cm^−2^ for the target‐ and control‐devices, respectively, derived from the external quantum efficiency (EQE) measurement, matched well with those obtained from the *J*‐*V* sweep measurement (Figure 4b). The synergistic effects arising from the NiO_x_‐mediated doping strategy were prominently manifested as a substantial increase in *V*_OC_ from 1.154 to 1.175 V. Furthermore, the target‐HTL‐integrated PSCs demonstrated substantial device‐to‐device reproducibility, as evidenced by a narrower performance distribution (Figure 4c and Figure S11), and exhibited a negligible hysteresis index (Table S2). Intriguingly, the photovoltaic parameters obtained through the NiO_x_‐mediated p‐doping strategy proved to be commensurate with those of PSCs fabricated with conventionally doped HTLs (39 µL *t*BP, 23 µL LiTFSI, and 10 µL FK209) (Figure S12 and Table S3). While the optimized reference PSCs delivered a peak PCE of 25.21%, our strategic doping approach afforded a slightly higher maximum PCE of 25.24%, accompanied by a distinctly narrower performance distribution, notwithstanding the complete exclusion of the *t*BP additive.

The enhanced conductivity of spiro‐OMeTAD, along with its energetically favorable alignment with the perovskite layer, are conducive to promoting efficient interfacial hole extraction and mitigating energy loss at the heterojunction. To comprehensively elucidate the impact of the NiO_x_‐mediated doping strategy on interfacial hole extraction kinetics, both steady‐state PL and time‐resolved PL (TRPL) measurements were employed on glass/perovskite/control‐ and target‐HTL configurations. The pristine glass/perovskite sample exhibited pronounced PL emission, whereas the subsequent deposition of spiro‐OMeTAD induced substantial PL quenching (Figure S13a). Particularly, the interfacial enhancements culminated in a markedly enhanced PL quenching under the target‐HTL condition, signifying the efficient extraction and transport of photoexcited charge carriers from the perovskite absorber. This interfacial charge transfer behavior was further corroborated by a significantly shortened PL lifetime observed under the same conditions (Figure S13b). Additionally, the markedly elevated recombination resistance (*R*_rec_), coupled with its attenuated bias dependence under the target‐HTL condition, underscores a substantial suppression of interfacial non‐radiative recombination pathways and a concomitant reduction in charge carrier accumulation at the interface [36, 37], thereby contributing to an appreciable enhancement in the *V*_OC_ (Figure S14).

Of greater significance, our strategic approach transcends mere efficiency enhancement, conferring exceptional long‐term stability even under rigorously demanding conditions, including MPPT operation and damp‐heat stress testing. Particularly, the NiO_x_ constitutes as efficacious avenue for circumventing stability liabilities, by virtue of its dual capacity to supplant *t*BP and sequester Li^+^ ions. Ultimately, the NiO_x_ powder, being removed during the filtration step, operates a transient catalytic facilitator throughout the p‐doping process, thereby yielding a chemically benign spiro‐OMeTAD solution. The observed solvation capability of NiO_x_ toward the LiTFSI dopant, coupled with its concurrent sequestration of residual Li^+^ ions, imparts enhanced resilience to device degradation by alleviating the adverse effects arising from the migration of ionic species, including Li^+^ ions and their associated byproducts within the device. In particular, such severe migration of ionic species, exacerbated under external perturbations, precipitates in their accumulation as electrically insulating byproducts [33, 38], thereby markedly compromising the charge transport characteristics of the spiro‐OMeTAD HTL. Moreover, this deleterious phenomenon concurrently instigates the incursion of iodide ions from the perovskite absorber (Figure S15), culminating in the progressive de‐doping of p‐doped spiro‐OMeTAD and a concomitant deterioration of its intrinsic electrical functionality [39].

Firstly, to elucidate the impact of a drastic reduction in Li^+^ ion concentration on the electrical characteristics of the spiro‐OMeTAD, we systematically evaluated the variations in the electrical conductivity of spiro‐OMeTAD under conditions of photoirradiation and external bias (Figure S16). The electrical conductivity of the control‐HTL underwent a substantial decrease, retaining only 55.8% of its initial value after 1,000 h of continuous light illumination. Moreover, the application of a 5 V external bias induced an irreversible junction breakdown, as evidenced by a reduction in the electrical conductivity, which catastrophically disrupted charge‐carrier transport within the device. In contrast, the target‐HTL demonstrated exceptional stability in its electrical characteristics under continuous photoirradiation and applied external bias, sustaining over 90% of its initial value after 1,000 h of continuous light exposure and maintaining over 80% under an applied external bias of 7 V. Leveraging the enhanced electrical stability of the target‐HTL, we quantitatively assessed the operational stability of PSCs under maximum power point tracking (MPPT) conditions with continuous 1‐sun illumination (Figure 4d). The PSCs incorporating the target‐HTL preserved over 90% of their initial PCEs after 500 h of continuous operation, whereas devices employing the control‐HTL exhibited a 71% decline in PCE within 500 h. Notably, the PSCs using conventionally doped HTLs suffered an 80% reduction in PCE following 300 h of continuous operation (Figure S17). Secondly, in the absence of the *t*BP additive in our targeted HTL formulation, the elevated glass transition temperature (*T*_g_) of the spiro‐OMeTAD doped via NiO_x_‐mediated strategy (Figure S18) conferred exceptional damp‐heat stability to the PSCs, enabling retention of 95% of their initial PCE under stringent accelerated aging conditions (85°C/85% relative humidity [RH]) (Figure 4e and Figure S19). To the best of our knowledge, the device lifetime measured under damp‐heat stress conditions constitutes the most outstanding value reported in the literature to date (Table S4). Notably, this lifetime even surpasses that of devices incorporating PTAA in place of spiro‐OMeTAD. Consequently, the proposed innovative doping strategy offers a holistic solution to the longstanding challenges associated with conventional spiro‐OMeTAD doping systems, while concurrently ensuring long‐term operational stability under rigorous thermal and humid conditions, thereby accentuating the promising trajectory of n‐i‐p structured PSCs for future advancement.

## 3 Conclusion

We put forward a paradigm‐shifting and transformative strategy that leverages the redox‐catalytic functionality of NiO_x_ to overcome the intrinsic thermal instability of spiro‐OMeTAD‐based HTL systems. The intrinsic redox activity of NiO_x_ facilitates the pre‐oxidation of spiro‐OMeTAD, triggering ultrafast hole injection and rapid formation of p‐doped radicals. Concurrently, the negatively charged surface sequesters Li^+^ ions from LiTFSI, promoting the dissociation of LiTFSI dopant even in the absence of *t*BP, and effectively removing the captured ionic species along with the reacted NiO_x_ powder during filtration. The resulting PSCs achieve an exceptional maximum PCE of 25.24% despite the absence of *t*BP, while demonstrating superior operational reliability under damp‐heat stress conditions for over 1,000 h, retaining 95% of their initial performance metrics. Beyond direct enhancements in device performance, this work establishes a new paradigm in dopant engineering by simultaneously addressing efficiency and long‐term stability. As such, it represents a significant advancement in the field of perovskite photovoltaics, offering a robust and forward‐looking strategy for the development of sustainable optoelectronic technologies.

## Author Contributions

Y.S.S., M.K., D.S.K., and S.A. conceived the idea and designed the experiments. Y.S.S., S.H., I.J., D.S.K., and S.A. wrote the manuscript. M.K. fabricated the PSCs and performed their long‐term operational stability measurements. J.L. performed long‐term damp‐heat stability measurements. C.H.Y. conducted XPS and UPS measurements and data analysis. J.S. conducted FT‐IR measurements and data analysis. G.‐C.C. and J.‐Y.S. conducted nano‐IR AFM measurements and data analysis. S.P. and S.C. conducted KPFM measurements and data analysis. M.J.S. conducted UV–vis absorption and PL measurements and data analysis. D.L. conducted EIS measurements and data analysis. M.S. conducted TRPL measurements and data analysis. K.S., J.B., Y.J., and J.Y.K. suggested additional experiments and offered feedback. All authors discussed the results and contributed to the final manuscript.

## Conflicts of Interest

The authors declare no conflicts of interest.

## Supporting information




**Supporting File**: advs73939‐sup‐0001‐SuppMat.docx.

## Data Availability

The data that support the findings of this study are available in the supplementary material of this article.;
