# Supplementary material for: Redox‐Active NiOx‐Catalyzed Li+ Capture‐Extraction Strategy for tBP‐Free Spiro‐OMeTAD Enables Exceptional Damp‐Heat Stability in Perovskite Solar Cells
Source: Adv Sci (Weinh). 2026 Jan 26;13(25):e21825. doi: 10.1002/advs.202521825 (PMC13137786; doi:10.1002/advs.202521825)
Supplement: Supplementary file 1 — Supporting File: advs73939‐sup‐0001‐SuppMat.docx. [file ADVS-13-e21825-s001.docx]

**Supporting Information**

**Redox-Active NiO_x_-Catalyzed Li^+^ Capture-Extraction Strategy for *t*BP-Free spiro-OMeTAD Enables Exceptional Damp-Heat Stability in Perovskite Solar Cells**

*Yun Seop Shin*,^1,2,3^ *Minjin Kim*,^4^ *Jaehwi Lee*,^2^ *Chang Hyeon Yoon*,^1^ *Jongdeuk Seo*,^2^ *Gyeong-Cheon Choi*,^5^ *Sujung Park*,^6^ *Min Jung Sung*,^1^ *Kyungnan Son*,^7^ *Sungjun Hong*,^7^ *Inyoung Jeong*,^7^ *Junseop Byeon*,^7^ *Yimhyun Jo*,^4^ *Dongmin Lee*,^1^ *Minseong Kim*,^1^ *Shinuk Cho*,^6^ *Ji-Youn Seo*,^5^ *Jin Young Kim*,^1,2^ *Dong Suk Kim*,*^1,2^ and *SeJin Ahn**^7^

^1^Graduate School of Carbon Neutrality, Ulsan National Institute of Science and Technology (UNIST), Ulsan 44919, Republic of Korea

^2^School of Energy and Chemical Engineering, Ulsan National Institute of Science and Technology (UNIST), Ulsan 44919, Republic of Korea

^3^Photoenergy Research Center, Korea Research Institute of Chemical Technology (KRICT), Daejeon, 34114, Republic of Korea

^4^Ulsan Advanced Energy Technology R&D Center, Korea Institute of Energy Research (KIER), Ulsan 44776, Republic of Korea

^5^Department of Nano Fusion Technology, Pusan National University, Busan 46241, Republic of Korea

^6^Department of Physics and Energy Harvest Storage Research Center, University of Ulsan, Ulsan 44610, Republic of Korea

^7^Photovoltaics Research Department, Korea Institute of Energy Research (KIER), Daejeon, 34129, Republic of Korea

*Corresponding author. kimds@unist.ac.kr and swisstel@kier.re.kr

**Experimental Section**

**Materials**

Fluorine-doped tin oxide on glass (FTO) was purchased from Asahi. Hydrogen peroxide (H_2_O_2_, electronic grade), ethanol (EtOH, 95%, special grade), and ethyl ether (99.5%, special grade) were purchased from SAMCHUN. Hydrochloride (HCl, 37 wt% in water) was purchased from Junsei Chemical Co. Methylammonium chloride (MACl, for synthesis), *N*,*N*-dimethylformamide (DMF, anhydrous 99.8%), dimethyl sulfoxide (DMSO, >99.5%), 2-propanol (IPA, anhydrous, 99.5%), 2-methoxyethanol (2-ME, anhydrous, 99.8%), acetonitrile (anhydrous, 99.8%), chlorobenzene (CB, anhydrous, 99.8%), potassium chloride (KCl, ACS reagent, 99.999% trace metals basis), urea (ACS reagent, 99.0-100.5%), thioglycolic acid (TGA, ≥99%), tin(II) chloride dihydrate (SnCl_2_·2H_2_O, ≥99.995%), lithium bis(trifluoromethanesulfonyl)imide salt (LiTFSI), 4-*tert*-butylpyridine (*t*BP, 98%), and ethylene carbonate (EC, 98%) were purchased from Sigma-Aldrich. Tin (IV) oxide (SnO_2_, 12% in H_2_O colloidal dispersion) was purchased from Xi’an Yuri Solar Co. Lead(II) iodide (PbI_2_, 99.99% trace metal basis) was purchased from TCI. Formamidinium iodide (FAI) and *n*-octylammonium iodide (OAI) was purchased from Greatcell Solar Materials. Spiro-OMeTAD (99.5%) and FK209 Co(III) TFSI salt (>99%) were purchased from Lumtec. Nickel oxide Ni_2_O_3_ powder was purchased from US Research Nanomaterials, Inc.

**Fabrication of PSCs**

The whole process of the PSCs fabrication was carried out at controlled dry room (20 ℃/20% RH). The perovskite precursor solution was prepared by mixing 1,202 mg FAPbI_3_, 35 mol% MACl, and 0.8 mol% MAPbBr_3_ in a mixture of DMF and DMSO (4:1). The filtered perovskite solution, using a 0.2 µm PVDF filter, was spread over the as-prepared SnO_2_ substrate at 7,500 rpm for 50 seconds with a ramping duration of 0.1 seconds. During the spin-coating process, 1 mL of diethyl ether, serving as an anti-solvent, was dripped after spinning for 13 seconds, followed by immediate annealing on a hot plate at 150 ℃ for 15 minutes. To passivate the surface of the perovskite, 4 mg/mL of octylammonium iodide dissolved in IPA was spin-coated on top of the perovskite film at 3,000 rpm for 30 seconds. After then, the hole-transporting layer was deposited by spin-coating a spiro-OMeTAD (Lumtech) solution, containing 7 µL of LiTFSI, at 4,000 rpm for 30 seconds. For the target conditions, 3 mg of NiO_x_ powder was incorporated into a spiro-OMeTAD solution (113.6 mg/mL in CB) containing 7 µL of LiTFSI (0.516 g/mL in ACN) and 10 µL of FK209 (0.375 g/mL in ACN). The resulting mixture was blended for 5 seconds and subsequently filtered prior to use. The deposition conditions for the spiro-OMeTAD layer were identical to those employed for the control spiro-OMeTAD solution. In this study, device performance was further refined to attain enhanced efficiency through the supplementary incorporation of the FK209 dopant, which facilitates more effective p-type doping. Finally, a gold electrode (80 nm) was deposited by thermal evaporation under a high vacuum of 10^–6^ Torr.

**Characterization of the spiro-OMeTAD and perovskite films**

UV-visible absorption spectra were measured using a spectrophotometer (Cary 5000, Agilent). ESR spectra were measured using a spectrometer (EMXplus, Bruker Optics). PL spectra were obtained using a fluorometer with xenon lamp as an excitation source (nF900, Edinburgh Instruments). XPS and UPS spectra were measured using a spectrometer (ESCALAB 250XI, Thermo Fisher Scientific) at a base pressure of 1.0×10^−9^ Torr with a monochromated Al-Kα X-ray source. FT-IR spectra were measured using a spectrometer with attenuated total reflection mode (670-IR, Varian). Surface potential was measured using a Kelvin probe force microscope (Nanocute, SII NanoTechnology Inc.). Time-resolved PL spectra were measured using a time-correlated single-photon counting (TCSPC) spectrometer (FluoTime 300, PicoQuant). The samples were photoexcited at 510 nm using a pulsed diode laser head (LDH-D-C-510, PicoQuant). The potential was measured using two independent lock-in amplifiers, with the cantilever resonance frequency serving as the feedback. The probe used was a Rh-coated Si cantilever with a resonance frequency of 25 kHz.

**ToF-SIMS measurements**

ToF-SIMS profiling measured the depth distribution of ions within the structure of FTO/SnO_2_/perovskite/spiro-OMeTAD/Au using a spectrometer (ToF-SIMS 5, ION TOF) equipped with a pulsed Bi^+^ cluster ion beam (25 keV and 1 pA). To accomplish the depth profiling measurement, Cs (0.25 keV and 12 nA) and O_2_ (0.5 keV, 88 nA) ion beams were utilized for negative and positive modes, respectively. The sputter size was 250 × 250 µm, while the analysis area for depth profiling was 50 × 50 µm.

**PiFM measurements**

PiFM measurements were performed using a Park Systems FX200 IR AFM equipped with quantum cascade laser (QCL) and operated in direct PiFM mode. In this mode, the photo-induced dipole-dipole interaction between the AFM tip and the sample is directly detected at the cantilever’s first resonance frequency, enabling localized infrared absorption mapping. Measurements were carried out under ambient conditions with a scan area of 10 × 10 μm^2^ and 1 × 1 μm^2^ at a resolution of 512 × 512 pixels, using a scan rate of 0.3 Hz. PiFM amplitude images were acquired at a selected wavenumber of 1508 cm^−1^, corresponding to the C–N stretching vibration of spiro-OMeTAD. Prior to measurement, the system was calibrated using the auto beam alignment procedure and the IR intensity was normalized based on a standard reference (SiO_2_ at 1100 cm^−1^).

**DMA measurements**

Glass transition temperature of spiro-OMeTAD was measured using a thermal analyzer (DMA Q850, TA Instruments) in stretch mode, with a maximum displacement amplitude of 15 µm, a vibration frequency of 1 Hz, and a heating rate of 3 ℃/min from 30 to 150 ℃. Samples were prepared using poly(propylene) (PP).

**Characterization of PSCs**

Unencapsulated PSCs were measured with a solar simulator (Newport-Oriel 94083A, Class AAA) in conjunction with a Keithley source meter 2400, under ambient conditions (25 ℃/20% RH). The light intensity was calibrated to AM 1.5G (100 mW/cm^2^) using a Si-reference cell certified by the National Renewable Energy Laboratory. The conventional *J*-*V* curves were measured under both forward (from a forward bias (−0.05 V) to a short circuit (1.25 V)) and reverse (from a forward bias (1.25 V) to a short circuit (−0.05 V)) scans with the fixed step voltage of 100 mV. To mitigate artifacts induced by scattered light, a non-reflective mask with an aperture area of 0.0803 cm^2^ was used to shield the active area of the device. EQEs were measured using a quantum efficiency measurement system (QUANTX-300, Newport Co.).

**Characterization of PSCs stability**

The damp-heat (85 ℃/85% RH) stability test was conducted using a temperature & humidity chamber (TH3-PE, Jeio Tech Co. Ltd). The performance of the devices was periodically assessed following their cooling to room temperature.


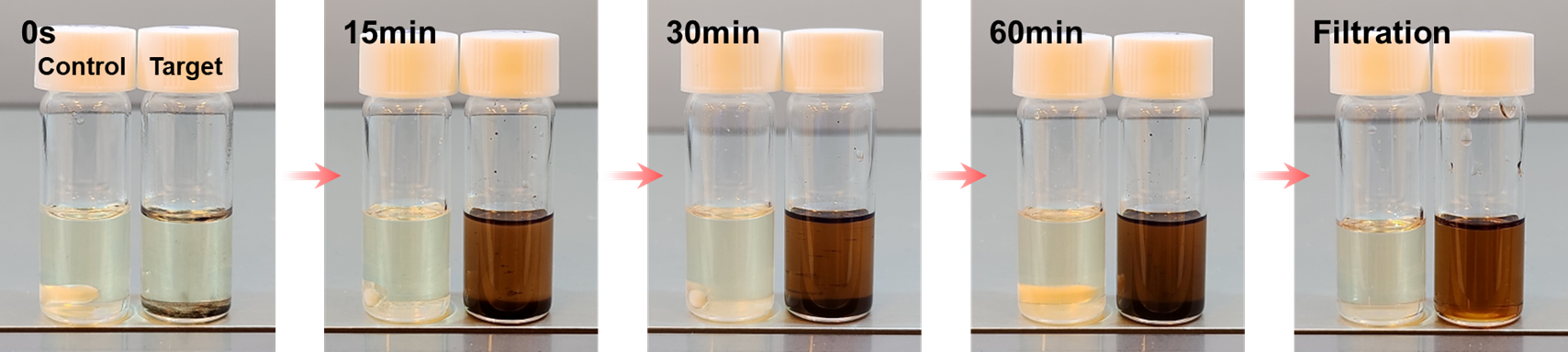


**Figure S1.** Photographic images of spiro-OMeTAD solutions under control and target conditions following light illumination.


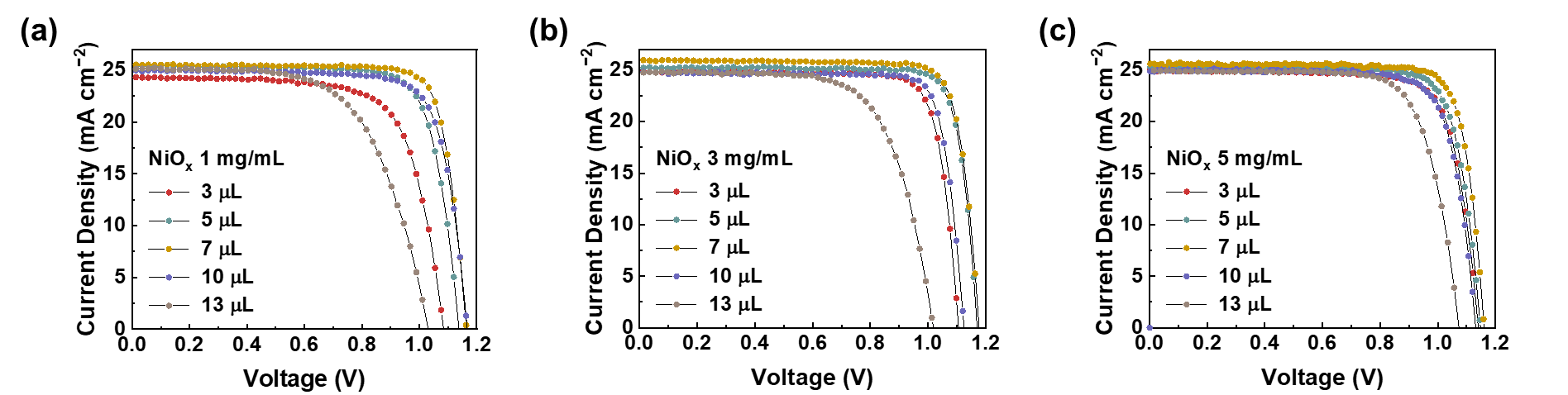


**Figure S2.** *J*-*V* characteristics of PSCs incorporating spiro-OMeTAD HTLs with varying concentrations of NiO_x_ and LiTFSI.


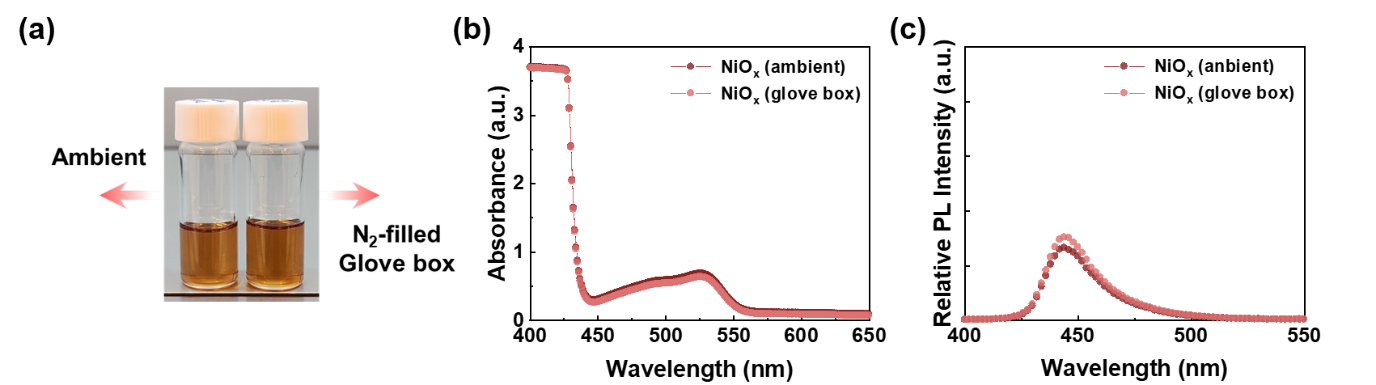


**Figure S3.** (a) Photographic images, (b) absorption spectra, and (c) PL spectra of spiro-OMeTAD solutions prepared under different conditions; ambient air and a N_2_-filled glove box.


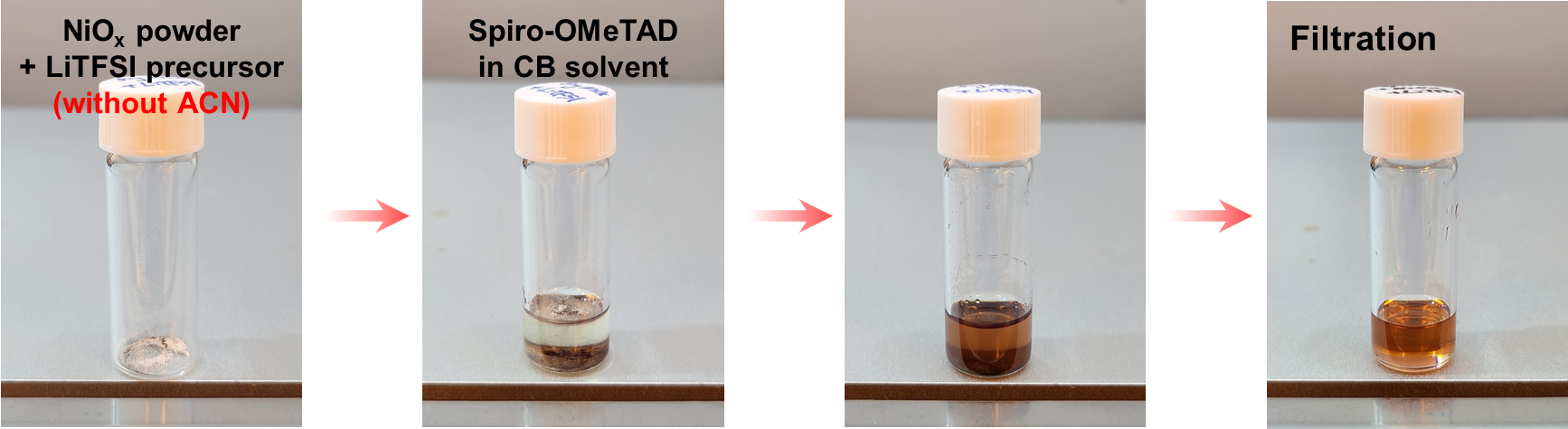


**Figure S4.** Schematic illustration of the NiO_x_-assisted p-type doping mechanism in the spiro-OMeTAD solution with direct addition of the LiTFSI precursor in the absence of ACN solvent.


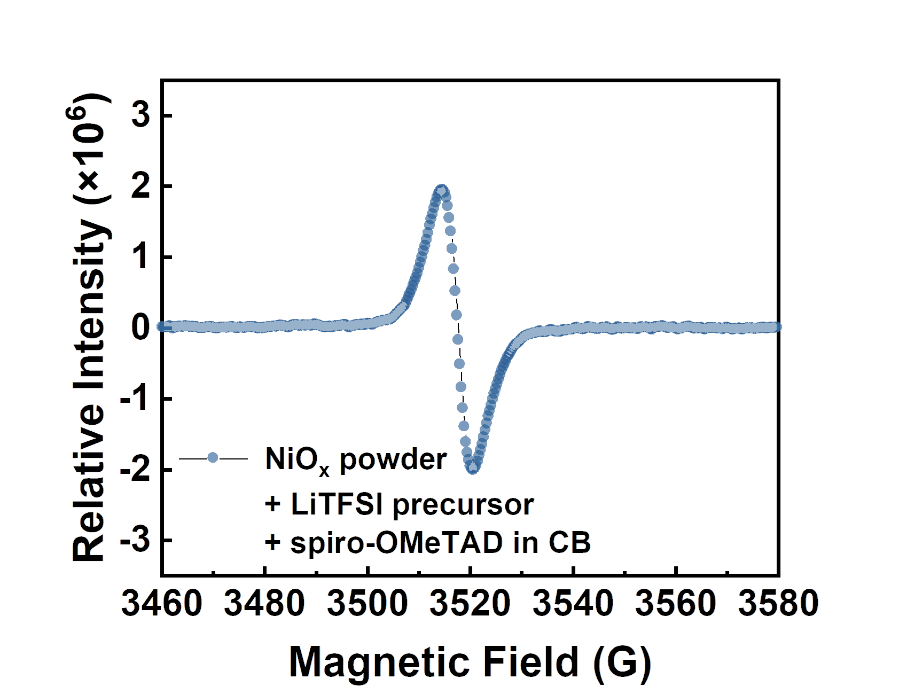


**Figure S5.** ESR spectra of NiO_x_-assisted p-doped spiro-OMeTAD solutions prepared by directly adding the LiTFSI precursor without the use of ACN solvent.


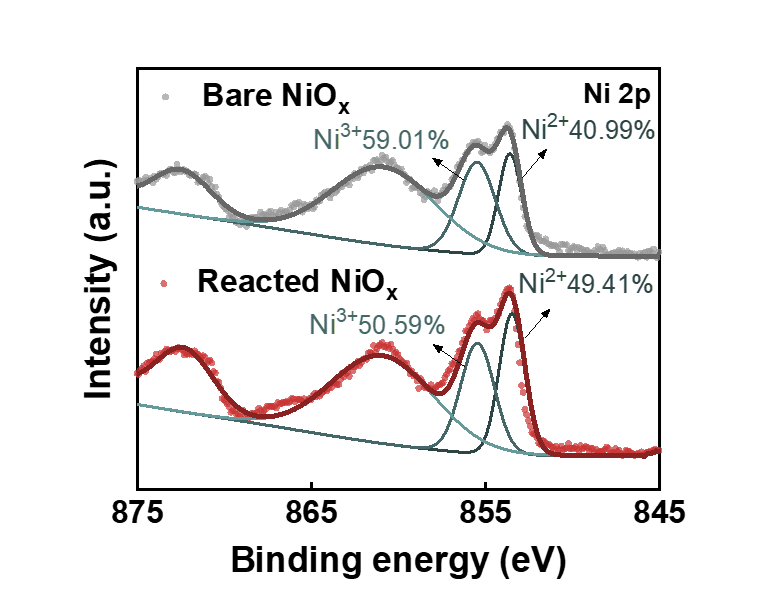


**Figure S6.** XPS spectra corresponding to Ni 2p for pristine and reacted NiO_x_ powders.


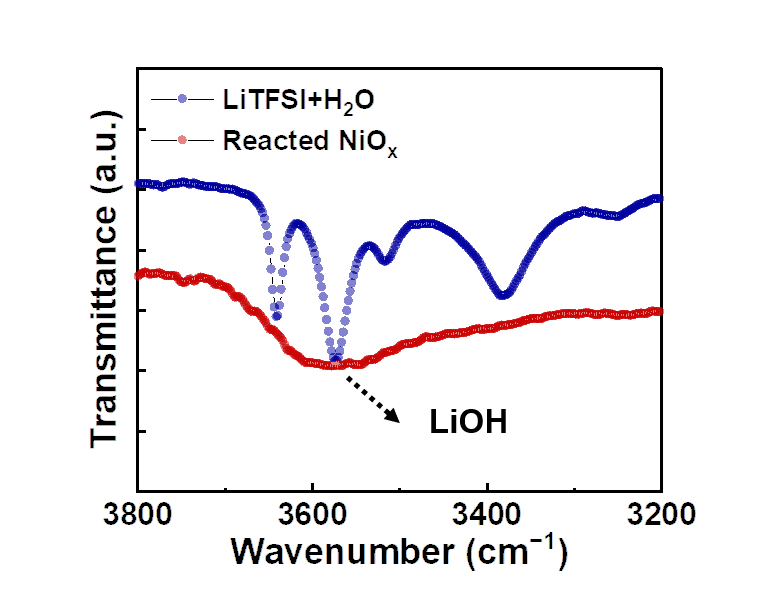


**Figure S7.** FTIR spectra of LiTFSI+H_2_O mixture and reacted NiO_x_ powder.


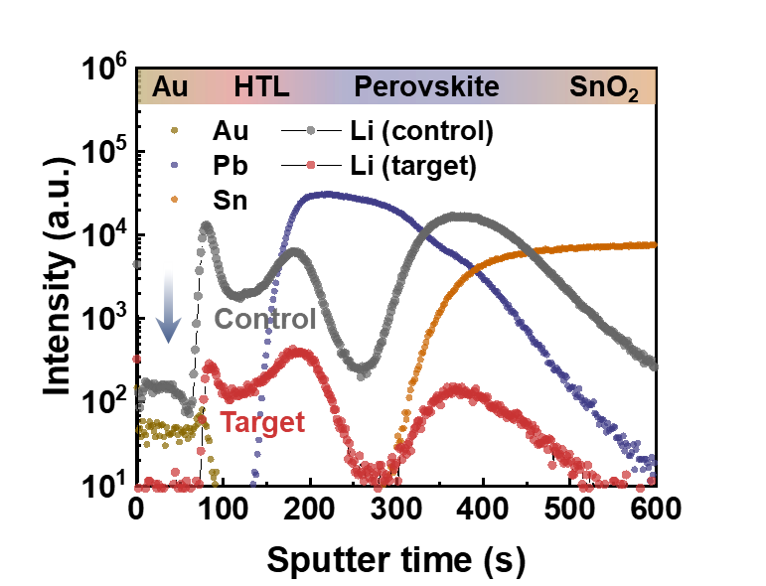


**Figure S8.** ToF-SIMS depth profiles of Li^+^ ions in device of FTO/SnO_2_/perovskite/control- and target-HTLs/Au.


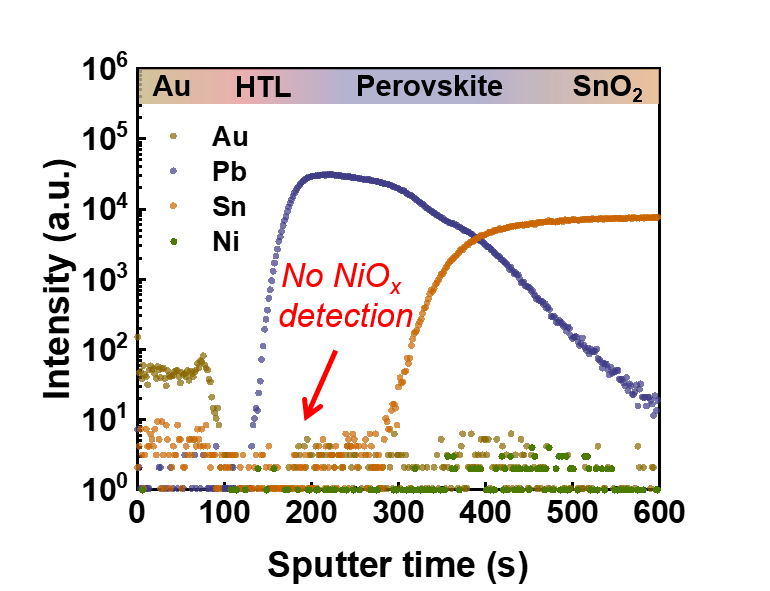


**Figure S9.** ToF-SIMS depth profiles of Ni ions in device of FTO/SnO_2_/perovskite/target-HTL/Au.


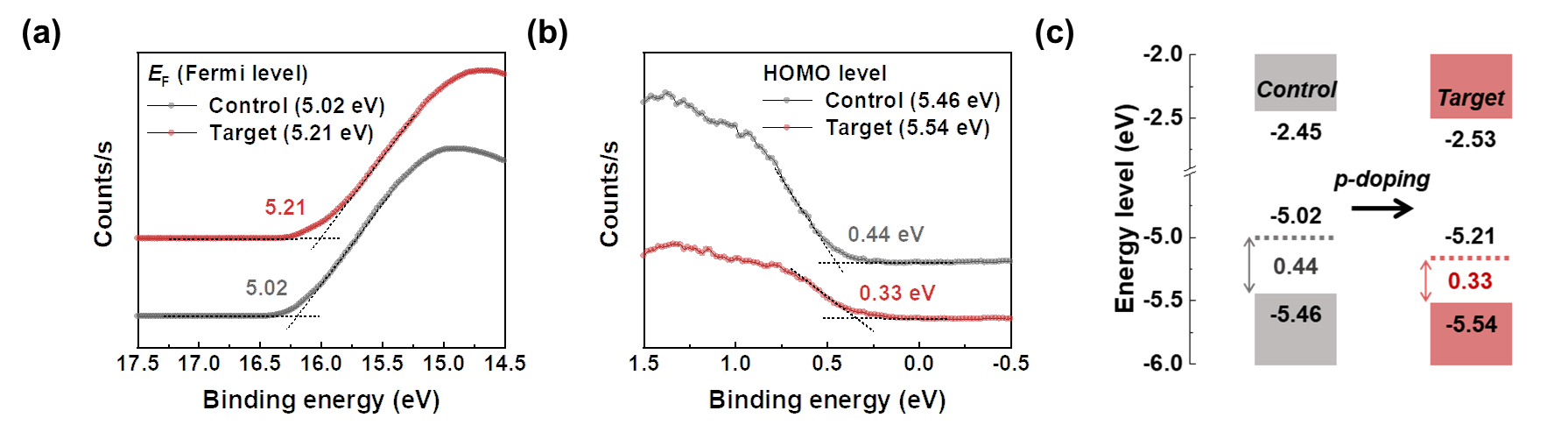


**Figure S10.** UPS spectra of the as-prepared control- and target-HTL films; (a) secondary edge region and (b) valence band edge plotted relative to a gold reference. (c) Schematic diagram of energy levels of control- and target-HTL films.


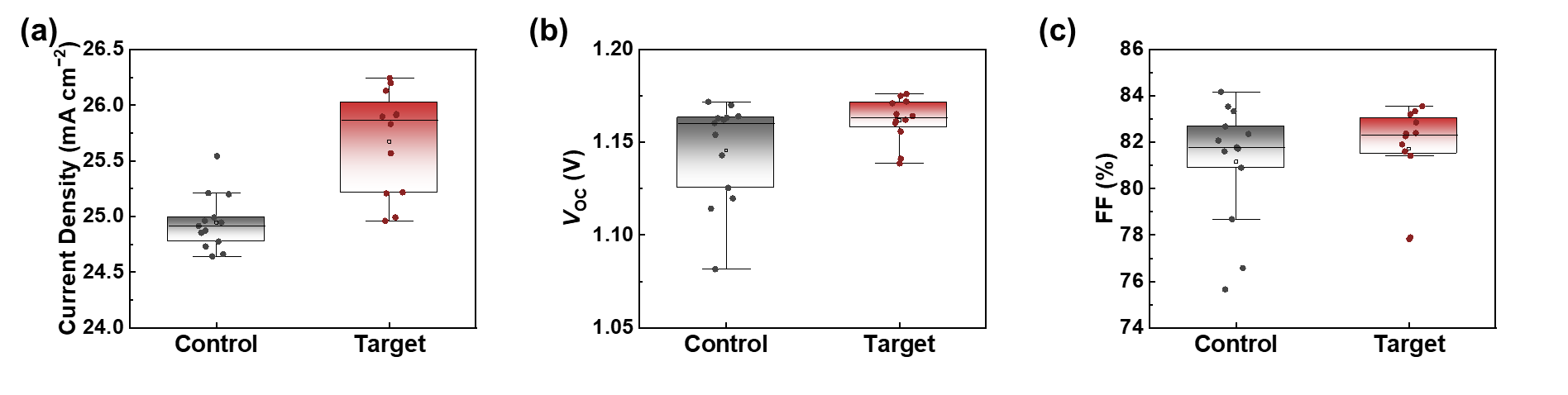


**Figure S11.** Statistical distribution of (a) *J*_SC_, (b) *V*_OC_, and (c) FF for the control- and target-PSCs.


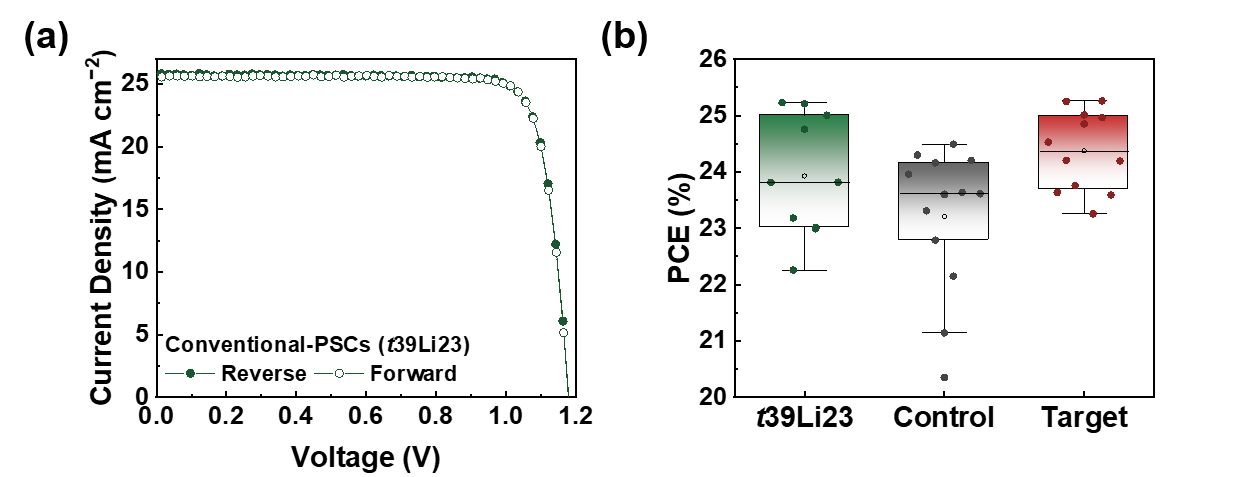


**Figure S12.** (a) *J*-*V* characteristics of PSCs incorporating conventionally doped HTLs (39 μL *t*BP, 23 μL LiTFSI, and 10 μL FK209) measured under both reverse and forward scanning directions. (b) Statistical distribution of PCE.


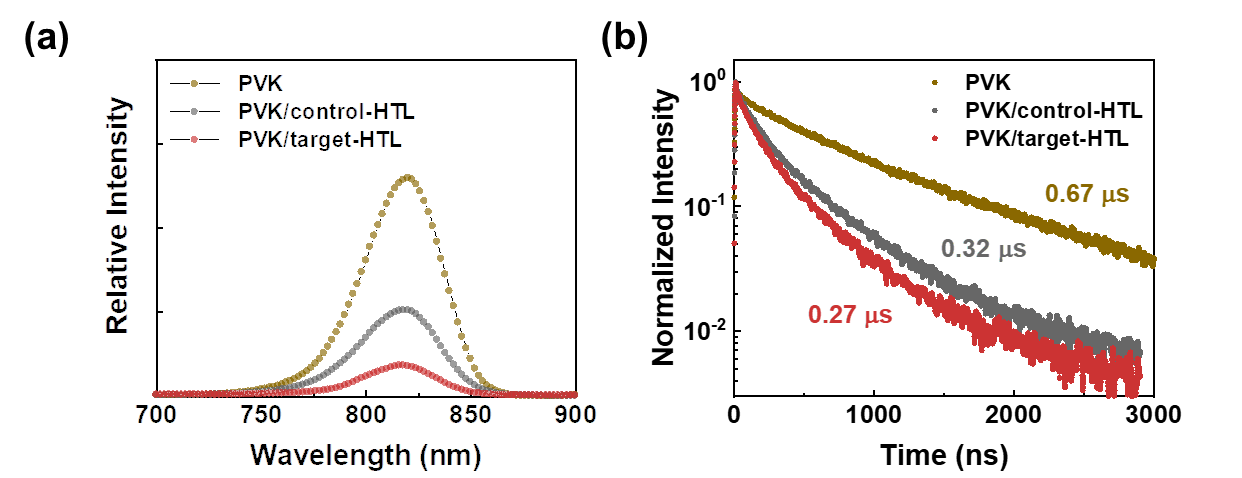


**Figure S13.** (a) Steady-state PL and (b) TRPL spectra of control- and target-HTLs deposited on the underlying perovskite layer.


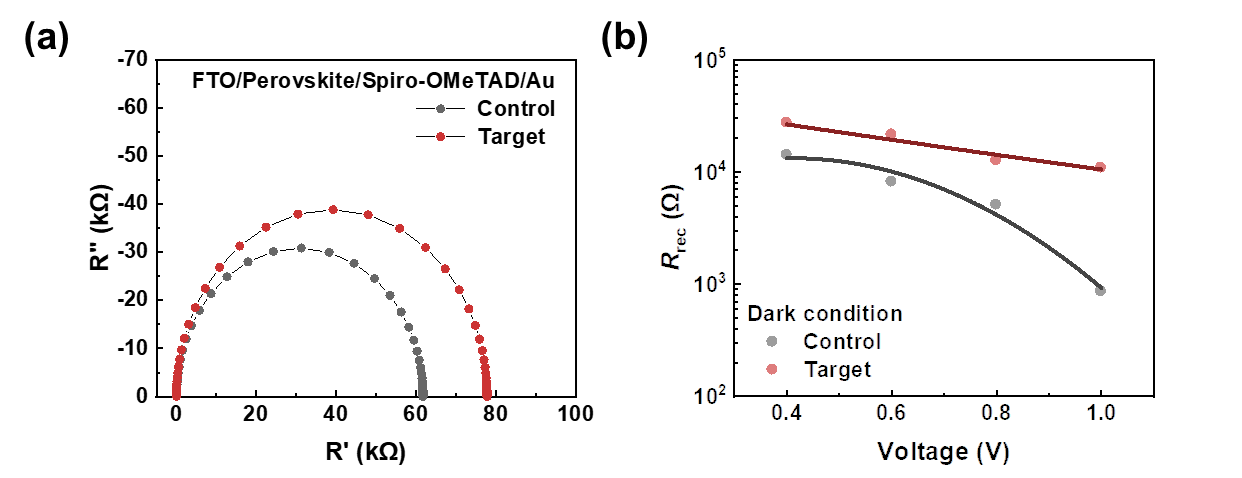


**Figure S14.** (a) Nyquist plots and (b) recombination resistance (*R*_rec_) of the PSCs using control- and target-HTLs.


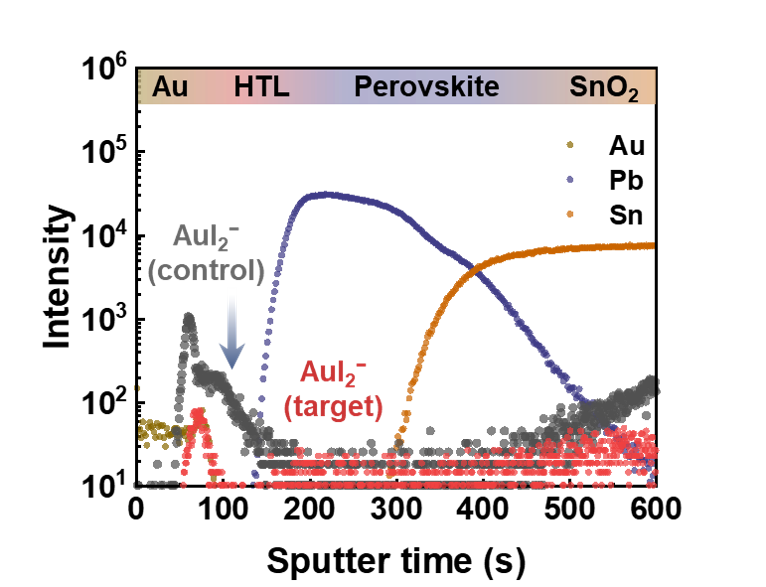


**Figure S15.** ToF-SIMS depth profiles of AuI_2_^–^ in device of FTO/SnO_2_/perovskite/control- and target-HTLs/Au.


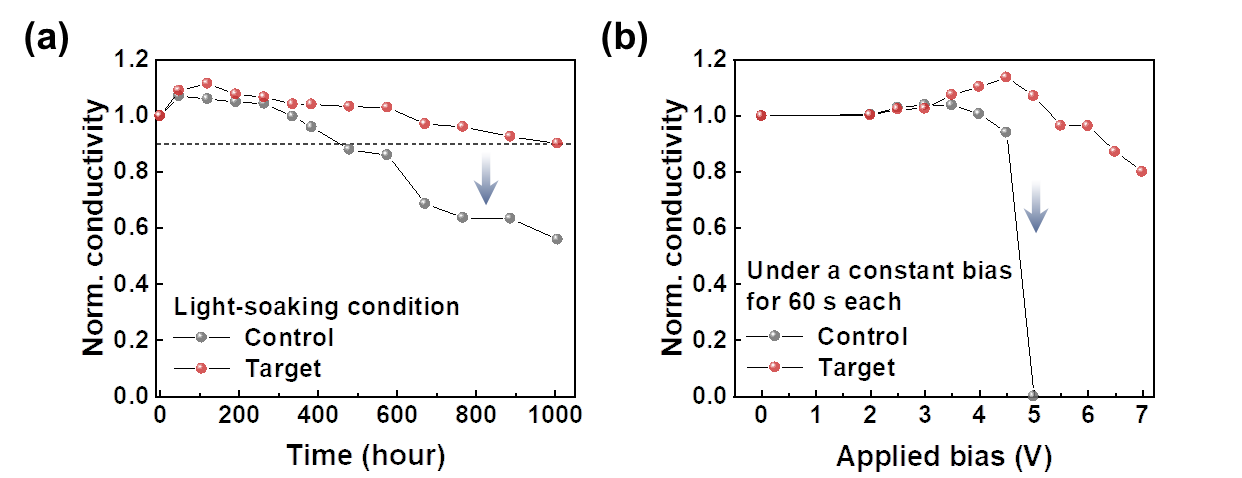


**Figure S16.** Variation of electrical conductivity of FTO/control- and target-HTLs/Au devices under (a) light illumination and (b) applied voltage.


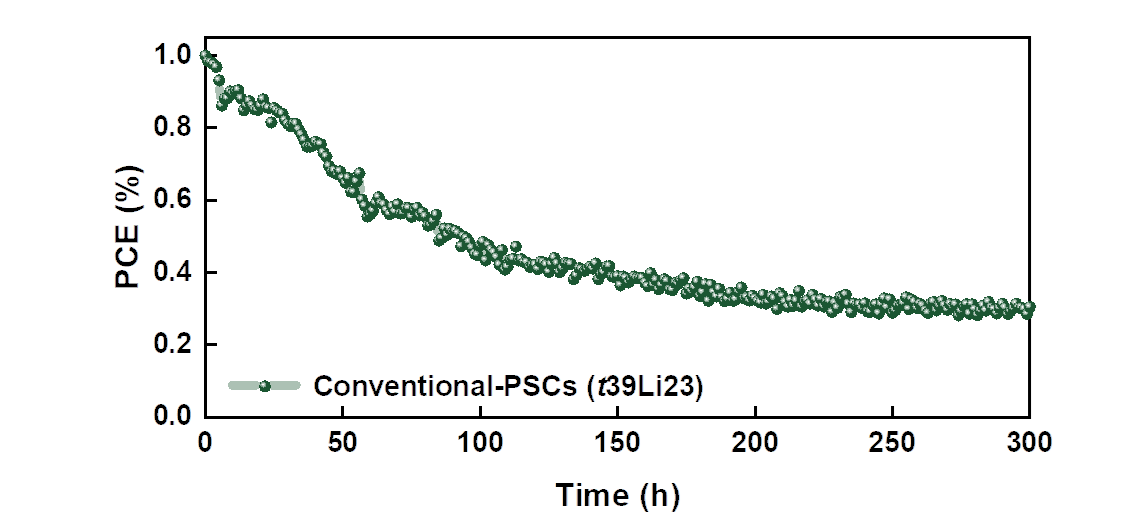


**Figure S17.** MPPT of encapsulated PSCs incorporating conventionally doped HTLs (39 μL *t*BP, 23 μL LiTFSI, and 10 μL FK209) under continuous 1-sun illumination.


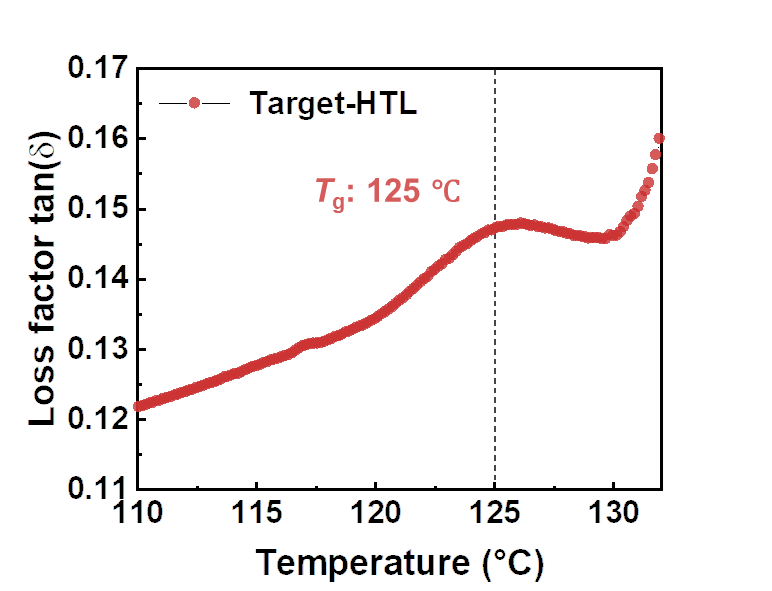


**Figure S18.** DMA graphs of NiO_x_-mediated doped spiro-OMeTAD films.


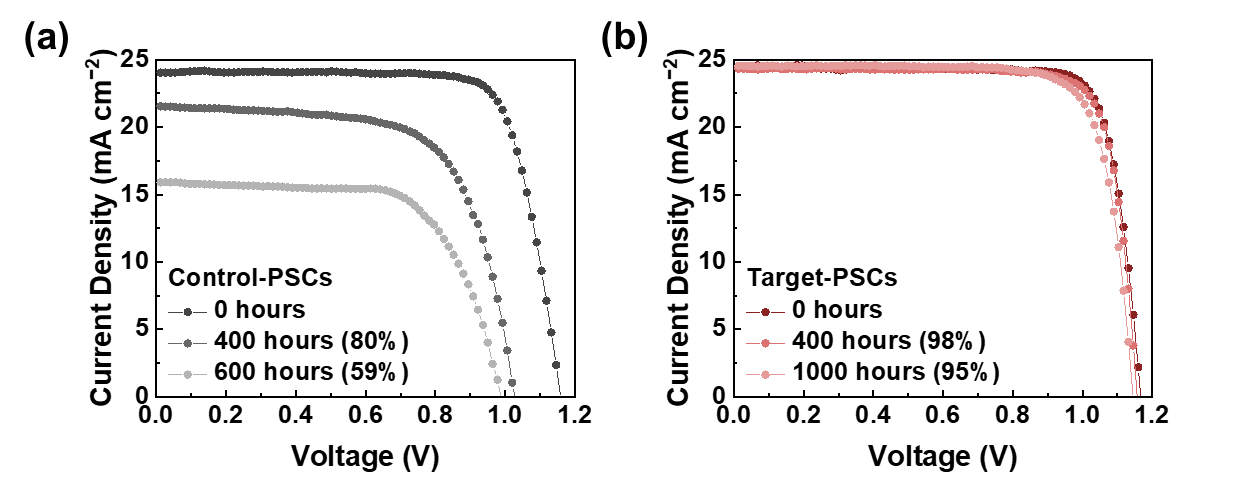


**Figure S19.** Variation of *J*-*V* curves of the control- and target-PSCs under the damp-heat (85 ℃/85% RH) conditions.

**Table S1.** Detailed *J*-*V* parameters of PSCs incorporating spiro-OMeTAD HTLs with varying concentrations of NiO_x_ and LiTFSI.

| **NiO_x_**  **(mg)** | **LiTFSI**  **(μL/mL)** | ***V*_OC_**  **(V)** | ***J*_SC_**  **(mA cm^−2^)** | **FF**  **(%)** | **PCE**  **(%)** |
| --- | --- | --- | --- | --- | --- |
| 1 | 3 | 1.084 | 24.32 | 71.38 | 18.82 |
|  | 5 | 1.138 | 25.40 | 77.95 | 22.53 |
|  | 7 | 1.164 | 25.45 | 82.03 | 24.30 |
|  | 10 | 1.167 | 24.92 | 78.16 | 22.73 |
|  | 13 | 1.032 | 25.18 | 62.63 | 16.27 |
| 3 | 3 | 1.106 | 24.83 | 81.67 | 22.42 |
|  | 5 | 1.170 | 25.28 | 83.61 | 24.74 |
|  | 7 | 1.175 | 25.83 | 83.21 | 25.24 |
|  | 10 | 1.124 | 24.71 | 83.52 | 23.21 |
|  | 13 | 1.018 | 24.71 | 68.42 | 17.21 |
| 5 | 3 | 1.141 | 24.96 | 77.76 | 22.16 |
|  | 5 | 1.148 | 25.26 | 79.91 | 23.18 |
|  | 7 | 1.161 | 25.57 | 81.86 | 24.31 |
|  | 10 | 1.132 | 24.89 | 78.53 | 22.12 |
|  | 13 | 1.074 | 24.85 | 74.80 | 19.96 |

**Table S2.** Detailed *J*-*V* parameters of PSCs incorporating control- and target-HTLs under both reverse and forward scanning directions.

|  | **Scan**  **direction** | ***V*_OC_**  **(V)** | ***J*_SC_**  **(mA cm^−2^)** | **FF**  **(%)** | **PCE**  **(%)** | **Hysteresis**  **index** |
| --- | --- | --- | --- | --- | --- | --- |
| Control | Reverse | 1.154 | 24.73 | 82.68 | 23.60 | 0.061 |
|  | Forward | 1.143 | 24.64 | 78.68 | 22.15 |  |
| Target | Reverse | 1.175 | 25.83 | 83.21 | 25.24 | 0 |
|  | Forward | 1.176 | 25.92 | 82.86 | 25.26 |  |

**Table S3.** Detailed *J*-*V* parameters of PSCs incorporating conventionally doped HTLs (39 μL *t*BP, 23 μL LiTFSI, and 10 μL FK209) under both reverse and forward scanning directions.

|  | **Scan**  **direction** | ***V*_OC_**  **(V)** | ***J*_SC_**  **(mA cm^−2^)** | **FF**  **(%)** | **PCE**  **(%)** | **Hysteresis**  **index** |
| --- | --- | --- | --- | --- | --- | --- |
| *t*39Li23 | Reverse | 1.180 | 25.82 | 82.77 | 25.21 | 0 |
|  | Forward | 1.179 | 25.57 | 83.74 | 25.23 |  |

**Table S4.** Comparative analysis of the damp-heat stability lifetime of PTAA- or spiro-OMeTAD-based PSCs.

| **Published**  **year** | **Conditions**  **(HTM)** | **Stability lifetime** | **Ref.** |
| --- | --- | --- | --- |
| 2023.03 | 85 ℃/85% RH  (PTAA) | *T*_90_ ~ 800 hours | ^[1]^ |
| 2023.11. | 85 ℃/85% RH  (PTAA) | *T*_83_ ~ 1,000 hours | ^[2]^ |
| 2024.02. | 85 ℃/85% RH  (Spiro-OMeTAD) | *T*_81.7_ ~ 500 hours | ^[3]^ |
| 2025.02. | 85 ℃/85% RH  (Spiro-OMeTAD) | *T*_85_ ~ 1,000 hours | ^[4]^ |
| 2025.03. | 85 ℃/85% RH  ( - ) | *T*_90.6_ ~ 500 hours | ^[5]^ |
|  | **85 ℃/85% RH**  **(Spiro-OMeTAD)** | ***T*_95_ ~ 1,000 hours** | **This work** |

**Supporting References**

1. Y. Kim, G. Kim, E. Y. Park, C. S. Moon, S. J. Lee, J. J. Yoo, S. Nam, J. Im, S. S. Shin, N. J. Jeon and J. Seo, *Energy Environ. Sci.*, 2023, **16**, 2226.

2. T. Wang, J. Yang, Q. Cao, X. Pu, Y. Li, H. Chen, J. Zhao, Y. Zhang, X. Chen and X. Li, *Nat. Commun.*, 2023, **14**, 1342.

3. H. Kim, J. Sin, M. Kim, G. Kim, M. Kim, J. Kim, G. Park, B. Kim, M. S. Jeong and J. Yang, *Sol. RRL*, 2024, **8**, 2300825.

4. Y. S. Shin, J. Lee, D. G. Lee, J. W. Song, J. Seo, J. Roe, M. J. Sung, S. Park, G. Y. Shin, J. Yeop, D. Lee, C. H. Yoon, M. Kim, J. G. Son, G.-H. Kim, S. Cho, J. Y. Kim, T. K. Lee and D. S. Kim, *Energy Environ. Sci.*, 2025, **18**, 3269.

5. C. Shao, J. Ma, G. Niu, Z. Nie, Y. Zhao, F. Wang and J. Wang, *Adv. Mater.*, 2025, **37**, 2417150.
